# Suicide across time and cultures: from a philosophical debate to network analysis

**DOI:** 10.1192/j.eurpsy.2024.1623

**Published:** 2024-08-27

**Authors:** C. Tapoi, R. Chancel, S. Baltzis, U. Cikrikcili, D. Cenci, J. Lopez Castroman

**Affiliations:** ^1^Department of Psychiatry, Dimitrie Gerota Emergency Hospital, Bucharest, Romania; ^2^Department of Emergency Psychiatry and Acute Care, Lapeyronie Hospital, CHU Montpellier, Montpellier, France; ^3^University Psychiatric Department, Helsingborg Hospital, Skåne, Sweden; ^4^Deutsche Zentrum für Neurodegenerative Erkrankungen, Magdeburg, Germany; ^5^Department of Transactional Medicine, Università del Piemonte Orientale, Novara, Italy; ^6^Department of Signal Theory and Communications, University Carlos III, Madrid, Spain

## Abstract

**Introduction:**

Suicide is a multifaceted subject that encompasses a broad spectrum of perspectives, spanning philosophy, the arts, social sciences, neuroscience, neuropsychiatry, and public health. The history of suicide is intricately intertwined with the history of humanity itself, and examining the shifting attitudes towards suicide holds significant implications for the field of suicide prevention.

**Objectives:**

The objective of this paper is to offer a timeline of the social perspectives about suicidal behavior throughout history in order to showcase the influence of cultural and contextual factors.

**Methods:**

This poster is based on the Massive Open Online Course (MOOC) “Focus on Suicidal Behaviour” provided by the European Psychiatric Association. We performed a brief overview of the chapter on history of suicide and updated data on this topic with recent literature findings.

**Results:**

In antiquity, suicide was sometimes regarded as justifiable, whether to preserve honor or protest injustices. However, during the Middle Ages, suicide was primarily seen as a criminal act, violating the rules of the Christian religion. The Renaissance brought about a shift in the perception of suicide, as it began to be depicted in art as a heroic or philosophical act. Moving into the Romantic period, suicide took on a tragic and noble connotation, often seen as an escape from unbearable suffering.

The 19th century marked a significant turning point when the social context started being recognized as a crucial factor in the development of suicidal behavior. In the 20th century, suicide was increasingly considered a public health problem. In the 21st century, the discourse on suicide has become multifaceted. On one hand, network analysis has enabled the development of an integrated model of suicide, emphasizing the complex interactions among various risk and protective factors. On the other hand, ethical and moral debates persist regarding assisted suicide and its indications.

This summary primarily centers on the historical context of suicide within Europe. However, attitudes toward suicide vary significantly across cultures. For instance, in China, suicide rates are higher for women than for men, while Japan has historically displayed a relatively tolerant attitude toward suicide, particularly within the military. In contrast, Islamic countries consider suicide a major sin and implicitly associate it with stigma.

**Conclusions:**

The understanding of suicide evolves over time and is deeply influenced by cultural contexts. Familiarizing ourselves with the historical perspectives on suicide is essential for comprehending this complex social and personal phenomenon. Such knowledge forms the foundation for the creation of effective prevention strategies.

**Disclosure of Interest:**

None Declared

